# Incidence and prevalence of violence toward health care workers in emergency departments: a multicenter cross-sectional survey

**DOI:** 10.1186/s12245-021-00394-1

**Published:** 2021-12-14

**Authors:** Mohammed Alshahrani, Razan Alfaisal, Khalid Alshahrani, Leyan Alotaibi, Hissah Alghoraibi, Eman Alghamdi, Lulwah Almusallam, Zaineh Saffarini, Salihah Alessa, Faten Alwayel, Lubna Saffarini, Ali Alrawdhan, Charlene Mapusao, Laila Perlas Asonto, Amal Alsulaibikh, Mohammed Aljumaan

**Affiliations:** 1 Department of Emergency, College of Medicine, Imam Abdulrahman Bin Faisal University, PO Box 40236, Al Khobar, 31952 Kingdom of Saudi Arabia; 2 Emergency Medicine, King Fahad Specialist Hospital, Dammam, Kingdom of Saudi Arabia; 3 College of Medicine, Imam Abdulrahman Bin Faisal University, Dammam, Kingdom of Saudi Arabia; 4 Emergency Medicine Department, Security Forces Hospital, Riyadh, Kingdom of Saudi Arabia; 5 Pediatrics Department, Dubai Health Authorities, Dubai, United Arab Emirates; 6 Emergency Medicine Department, King Fahd Medical City, Riyadh, Kingdom of Saudi Arabia; 7 Emergency Medicine, Rashid Hospital Trauma Center, Dubai, United Arab Emirates

**Keywords:** Emergency Department, Workplace violence, Health care workers

## Abstract

**Background:**

Workplace violence and abuse in the emergency department (ED) has increasingly become a serious and alarming phenomenon worldwide where health care professionals are more prone to violence compared with other specialties.

**Aims:**

We aimed to estimate prevalence, and types of work place violence made against health care workers (HCW) in emergency departments of Arabian Gulf area.

**Methods:**

We performed a descriptive cross-sectional study at several emergency departments in Saudi Arabia and United Arab Emirates wherein a previously validated questionnaire was distributed among health care workers. The survey consisted of 22 questions that assessed respondent’s workplace violence and/or abuse encounters, protective measures, available work place policies, and actions implemented to detect and deal with violence and abuse against healthcare providers. Descriptive statistics was used and *P* value < 0.05 was considered significant for all statistical tests performed.

**Results:**

Seven hundred HCW in eleven emergency departments agreed to participate in the survey. Four hundred ninety-two completed the questionnaire with a response rate of 70%. More than 90% of the respondents were in the 20–39 years old bracket with an approximately equal gender distribution. Then, 20.9% of the respondents stated that they were physically attacked and 32.3% were attacked with a weapon. Most of the respondents (75.6%) reported that they were verbally abused or bullied by patients or relatives of patients. Staff responses to emotional abuse varied among respondents with the most frequent response of “told the person to stop” (22%), followed by “took no action” (19%). Further, 83.3% of respondents stated that there was an existing policy and procedure guidelines for reporting work place violence while 30.1% reported that they had not used any of these measures.

**Conclusion:**

Workplace violence among HCW in the emergency departments are common in the Gulf area and can be serious in as far as use of weapons. Staff awareness focus on this under reported issue, and staff training to recognize and report potential aggression can predict a significant reduction of incidents.

## Introduction

Workplace violence has become a serious and an alarming phenomenon in the health care sectors across healthcare professions. Emergency departments and psychiatric settings are more prone to violence compared to other specialties [1]. Workplace violence has number of definitions, some of which are limited to physical assault or harm [2], and others limited to non-physical abuse such as verbal, emotional, or racial harassment [3]. According to WHO, work place violence is defined as an “ intentional use of power, threatened or actual, against another person or against a group in work-related circumstances, that either results in or has a high degree of likelihood resulting in injury, death, psychological harm, maldevelopment, or deprivation” [4].

It is difficult to estimate the true incidence and prevalence of violence against emergency health care professionals since there are different definitions of workplace violence, reporting system variations between different geographical areas, and lack of reporting mechanisms [1]. However, a surveillance survey done by Emergency Nurses Association in the UK showed that most cases of violence were verbal abuse against ER nurses (54.5%), wherein physical violence rarely occurred without verbal abuse (0.8%). In addition, the most prevalent types of physical violence are being grabbed or pulled (48.3%), while yelled and being shouted at were the most common form of verbal abuse (89.0%) [5]. Another study done in Kuwait to determine the prevalence of workplace violence against doctors in the ED revealed that 86% of physicians experienced verbal insults or imminent threat of violence, 28% experienced physical attacks, and 7% had experienced physical assaults which is more likely to cause serious or fatal injuries [6]. Despite limited available studies and information in Saudi Arabia, a study conducted in Tabuk showed that workplace violence was significantly higher against physicians (96.8%) followed by nurses (90.9%), particularly those who have longer experience in ED. Approximately 58% of violent insults during working hours took place during night shifts. According to the results, most cases of physical violence were committed by hands or fists (79%), whereas weapons or instruments were used in 17.3% of cases. Verbal violence was reported in only 38% of cases, which might be due to under reporting of cases [7].

Several risk factors are associated with increased incidence of workplace violence. Individual factors such as being a female healthcare worker, working in a foreign society with different cultures and habits, or dealing with mentally ill, alcoholic, or drug abuser patients [8, 9]. Environmental work place factors, for example, inadequate security on site, easy accessibility of dangerous objects like weapons and sharp objects, and shortage of staff during shifts that causes increased patient waiting times, may all lead to increased risk of violence [10]. In addition, the exposure to workplace stressors may increase the risk of aggression and abuse. For example, work overload, individual working, low supervisor or team support, insufficient communication between staff members, and working in area with high crime rates [11, 12]..

Violence at work can give rise to a range of physical and emotional outcomes. The effects of violence are varied and usually depends on the frequency, severity, and the violence type. Physical injuries for example, range from bruises to broken bones. On the psychological aspect, most of healthcare workers reported different emotional disturbance, ongoing fear, anger, depression, anxiety, and sleep disturbance [13, 14]. Moreover, the physical and verbal insult can negatively affect the worker career, and unfortunately most of the victims reported their plan to leaving their jobs [15].

The most common action that took place to reduce violence against healthcare workers is training on how to minimize, manage, and deal with violence [16]. However, there is no information about effectiveness of actions, policies, and programs which are set for preventing violence.

Several studies have been conducted in Saudi Arabia regarding the prevalence of violence against healthcare professionals in emergency departments over many years. However, none of these studies assessed the policies and actions that were implemented to detect violence toward healthcare providers working in the ED. Thus, to the best of our knowledge, this study is the first to include multiple centers to estimate the incidence and prevalence of violence against ED health care workers in the Arabian Gulf area, and to assess current policies and actions made to protect them. It is therefore anticipated that it will offer unique perspective on the subject.

## Materials and methods

This is a descriptive cross-sectional study aimed to estimate the prevalence and types of workplace violence made against health care workers in the emergency departments; we will also evaluate existing hospital policies and actions implemented to identify and deal with the situation or lack of reporting mechanisms thereof.

The study was conducted at 11 emergency departments in major hospitals from different geographical areas in the Kingdom of Saudi Arabia and United Arab Emirates [7 in Saudi Arabia (3 in the Eastern region, 3 in the middle region, and 1 in western region) and 4 in the United Arab Emirates (Dubai and Ajman Emirates)]. We included all health care providers working in the emergency department for more than 6 months who directly handles or attends to patients and their families. The health care providers included physicians, nurses, allied health care practitioners, and administrative or clerical staff of all nationalities and races. We excluded health care providers who are rotators, medical students and interns, and newly hired staff who spent less than 6 months working in the ED.

Authors modified and pilot tested a validated questionnaire adopted from UNISON, the Irish Nurses Organization and the Royal College of Nursing (UK) according to study objective. The questionnaire underwent pilot testing by 8 ED Consultants at King Fahd Hospital of the University, Saudi Arabia where feedback on accuracy, clarity, and ease of use was established. We used paper-based questionnaire and it was distributed and explained to all respondents manually by co-investigators (which are residents and medical interns) in different hospitals and regions between November 2017 and March 2018.

### Data management and analysis

Descriptive statistics was used to assess the baseline demographics and socioeconomic factors. Categorical variables were presented as frequencies and percentages while the numerical variables are presented as mean ± standard deviation. Student’s *T* test and one-way ANOVA analysis was used to check for significance between the multinomial demographics and the incidence of workplace violence. *P* value < 0.05 was considered statistically significant for all the statistical tests. SPSS version 22 was used for all data analysis. We used convenient sampling technique.

## Results

### Descriptive analysis

Among 700 health care providers and administrators working in the ED, 492 completed the questionnaires with a response rate of 70%. More than 90% of the respondents, working in the ED, were in the age group 20–39 years old, with approximately equal gender distribution. The majority of the respondents were physicians (54%) followed by nurses (34%), and other categories (12%). About 77% of the respondents had 1–15 years of work experience in the health sector (Table 1).

**Table 1:** Demographics

	Frequency	Valid percent
Age (*N* = 492)		
≤ 19	1	0
20–24	86	17
25-29	194	39
30–34	120	24
35–39	48	10
40–44	13	3
45–49	8	2
50–54	5	1
≥ 55	8	2
No answer	9	2
Gender (N= 492)		
Male	240	49
Female	234	47
No answer	18	7
Marital status (*N* = 492)		
Single	216	44
Married	238	48
Living with partner	1	0
Separated	19	4
No answer	18	4
Professional group (*N* = 492)		
Physician	263	54
Nurse	168	34
Allied Health staff	40	8
Administrative/clerical	6	1
Other Support staff	10	2
No answer	5	1
Years of work experience (*N* = 492)		
Under 1 year	82	16.7
1–5	193	39.2
6–10	134	27.2
11–15	51	10.4
16–20	19	3.9
> 20	13	2.6

Then, 94.3% of the respondents expressed direct interaction with patients during their routine work. Approximately 20.3% of participants responded that they were not worried about workplace violence, vs. 14% who were very worried. With regards to workplace policies and regulations, 83.3% stated that there was a procedure for reporting workplace violence and only 30.1% of the respondents reported that they had not the ability to use any of these procedures. Approximately 29.3% reported lack of encouragement to report workplace violence, while 70.7% stated that they were encouraged to report by employer (74.2%) or colleagues (23.3%).

Further, 20.9% of the respondents stated to be physically attacked in their workplace and 75.6% reported being verbally abused or bullied. The majority (89%) were abused by either patients or relatives of patients; among those, 32.3% were attacked with a weapon. 5.6% responded to being physically attacked by a staff member, 1.9% by general public, and 1 reported that he was attacked by his/her supervisor/management. The response to the incident among subjects varied considerably, where the most frequent responses were “pretended it never happened,” “sought help from union,” and “told a colleague.” When the subjects were asked if the violence could be prevented, more than half of them answered “yes.” About 77.3% of the victims were not injured vs. 22.7% injured. Among those who were injured, 48% required formal treatment, while taking time off from work after being attacked was reported by 30.9% of respondents. Among those, 91.6% took 1–3 days off work, and the consequences for the attackers were reported as “None” by 31.8%, “Reported to police” by 22.7%, “Don’t know” by 22.7%, and only 13.6% reported the consequences as received “Verbal warning.” Almost half of the respondents reported that they witnessed physical violence in their work place. Among those, 48.3% witnessed violence 2–4 times in the last 12 months, followed by 36.4% who witnessed only once. However, two respondents reported that they witnessed daily violence in their work place. Reporting an incident in the last 12 months (witnessed or experienced) was reported by only 27.5%. Among those, 55.6% reported that they had been disciplined for reporting an incident of workplace violence. Some respondent has the impression that reporting violent incidents would initiate a lengthy process of investigations intra-hospital or in extreme cases, at police stations. This would cause interruption to their work schedules and would add commitments in attending subsequent meetings for questioning on the incident; this has been considered in their answers as disciplined.

**Fig. 1:**
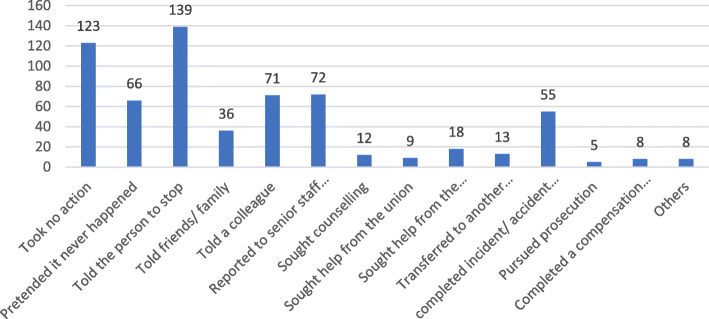
Responses to the emotional abuse among the study sample

Most of the respondents (75.6%) reported that they were verbally abused or bullied in the workplace. Among those, 8.5% reported “All the time,” 65.8% reported “Sometimes,” and 25.7% reported “Once.” The majority were abused by either patients or relatives of patients. Responses to the abuse varied among respondents with the most frequent response “Told the person to stop,” followed by “Took no action.” More details about the responses to the emotional abuse are illustrated in Fig. 1.

**Fig. 2:**
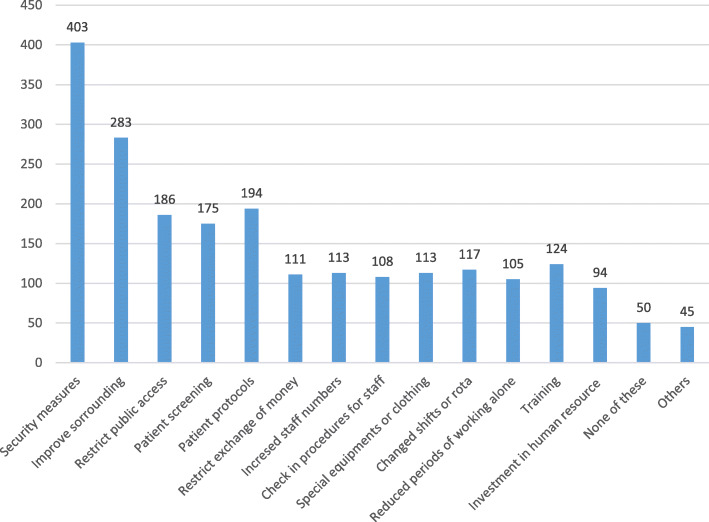
Responses of the study sample to the existing measures dealing with violence

**Fig. 3:**
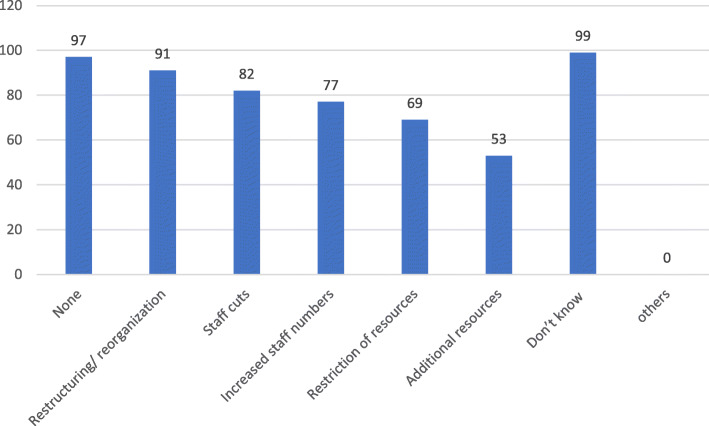
Changes occurred in the last 2 years in workplace

**Fig. 4:**
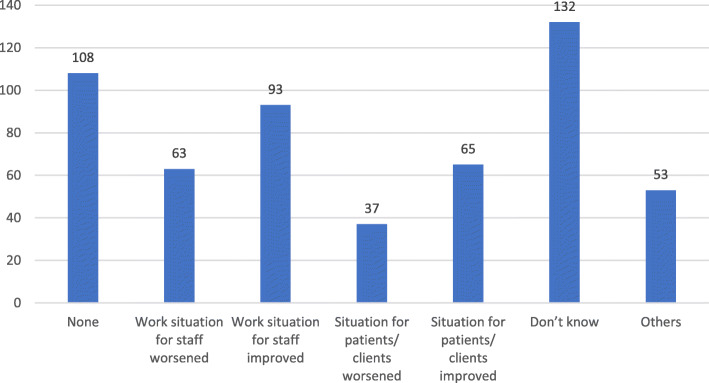
Impact of the changes on daily workplace

More than 70% of the respondents stated that the incident could have been prevented while only 16.4% reported that there was action taken to investigate the cause of the violence. Most of these actions were taken by the management/employer (91.5%). However, the consequences for the abuser were reported as “None” by 40.5%, “Verbal warning” by 28.6%, and “Reported to police” by only 4 respondents (9.5%). Most of the participants reported “Yes” on the development of specific policies on different categories in workplace except “Bullying/Mobbing” where most responded “Didn’t know” if there are specific policies regarding this issue (Table 2). With regards to existing helpful measures to deal with workplace violence, the most frequent response was security measures (73.9%) and improve surrounding or safer area for workspace (61.6%) (Fig. 2). Regarding the changes that have occurred in workplace in the last 2 years, the most frequent answer was “Don’t know” followed by “None” and then “Restructuring/Reorganization” while the least frequent answer was “Additional resources” (Fig. 3). The impact of those changes on daily work was varied clearly. Improvement for staff was the most selected choice, followed by improvement for patients (Fig. 4).

**Table 2:** Responses of the study sample to specific policies in workplace (%)

	Yes	No	Don’t Know
Health and safety	276 (69.5)	53 (13.4)	68 (17.1)
Physical workplace violence	292 (62.4)	76 (16.2)	100 (21.4)
Verbal abuse	209 (44.6)	96 (20.5)	164 (35.0)
Sexual harassment	205 (44.0)	79 (17.0)	182 (39.1)
Racial harassment	189 (40.6)	102 (21.9)	175 (37.6)
Bullying/ mobbing	175 (37.6)	90 (19.4)	200 (43.0)
Threat	205 (43.9)	89 (19.1)	173 (37.0)

Differences in responses by participant’s age brackets were highly significant (*P* < 0.001) in three questions (interact with patients, worried about violence in workplace, and physically attacked in workplace). Significant differences were also found according to age groups in responses to “Witnessed violence in workplace” (*P* = 0.028), “Reported an incident” (*P* = 0.001), “Who abused you” (*P* = 0.010), and “Investigation of the causes of the abuse” (*P* = 0.047). Regarding gender, significant differences were found only in two questions (Procedures for reporting violence, *P* = 0.009; and Witnessed physical violence, *P* = 0.048). Differences according to the marital status were found to be significant in four questions (Worried about violence, *P* = 0.012; Encouragement to report the violence, *P* = 0.049; physically attacked in workplace, *P* = 0.029; and Reported an incident, *P* = 0.009). The other differences were not significant. When the responses were related to the professional groups, there were highly significant differences (*P* < 0.001) between groups in responses to “Interact with patients” and “Being verbally abused or sexually harassed.” Significant differences were also found between groups in responses to “Worried about violence in workplace” (*P* = 0.009), “Encouragement to report violence” (*P* = 0.001), number of the staff in the same work (*P* = 0.002), “Who abused you” (*P* = 0.038), and “Investigation of the causes of the abuse” (*P* = 0.021). Differences in responses according to the present position were found to be highly significant (*P* < 0.001) in relation to “Interact with patients” and “Being verbally abused or sexually harassed.” In addition, significant differences in relation to “Reported an incident” (*P* = 0.008), “Times being emotionally abused” (*P* = 0.019), “If the incident could have been prevented” (*P* = 0.023), and “Investigation of the causes of the abuse” (*P* = 0.007). No significant differences for the remaining questions were observed (*P* > 0.05). With regard to experience, there were highly significant (*P* < 0.001) responses to “Worried about violence in workplace” and “Being verbally abused or sexually harassed”. Moreover, significant differences were also found in responses “Procedures for reporting violence” (*P* = 0.020), “Physically attacked in workplace” (*P* = 0.011), “Witnessed physically violence” (*P* = 0.020), and “Reported an incident” (*P* = 0.001). No other significant differences for the remaining questions were found.

In general, significance in differences was found only in two questions when the responses were related to gender, in four questions when related to marital status, in six questions when related to present position and experience, and in seven questions when related to age group and professional group. Most significant differences were related to the questions “Violence in workplace” and “Reported an incident.”

## Discussion

The results of this study raised concerns of violence against staff working in the emergency departments in this region, where 75.6% reported that they were physically and verbally attacked by either patients or patient’s relatives. Our findings echo other studies concerned with ED violence with similar situations met by physicians, nurses, and technical staff around the world [17–21].

Among the respondents, most of them were involved in direct interaction when they were working with adult patients and, to a lesser degree, elderly, then adolescents, and newborns.

Also, despite most respondent’s awareness to report workplace violence, higher portion lack the ability to report such situations. This result is not only seen in our study, but in another Arabian study (Albashtawy 2015) which stated that these violent actions were not reported by most of the victims to the responsible authorities [18].

Presence of other people, especially employer, to encourage respondents in reporting workplace violence shows positive impact to respondents, who reassure them to report violent situation.

Obviously, physical attack poses significant threat during a violent situation. However, respondents, in most cases, were assaulted without a weapon. Also, Al-Maskari (2020) stated similar interesting information regarding the higher frequency of verbal abuse toward ED nurses, in particular, during weekends [19]. One Turkish study appears to have a different opinion about the perpetrators of violence, as their study showed perpetrators were patients’ families and not the patients themselves [22].

According to British crime survey, various reasons that may affect the person’s ability to report violent actions were a mere sense of duty and a necessity to report any violence. While those who did not report took the violent matter lightly or was only perceived as a trivial thing [8].

The stress following the attack were frequently relieved either by “pretend it never happened” or “told a colleague,” with respondents having shared feelings that violence could have been evaded and prevented in the first place. Nonetheless, there are cases in which investigations have been done by management/employer to detect the cause behind the violence. It seems that Leary (2015) had a different opinion in his study that people are motivated, at minimum, to send a direct signal that they are aware of the violations and will not allow such actions in the future [17].

Fortunately, injuries were inflicted only to minority of the respondents, but, approximately, half of them needed to be treated. Also, a minority of respondents subjected to violence seemed to experience these situations for the first time, necessitating time off from work that did not exceed 2–3 days, which is similar to what British crime survey reported [8]. The repeated violence incidences place the respondents in a dilemma whether to report and be disciplined, or neglect and be always the victim.

Verbal abuse, sexual harassment, and bullying in workplace were experienced by respondents in varying degrees, and frequently met by only telling the person to stop or to take no action. Regarding frequency of abuse, physical abuse (27.6%) was always reported in the second place after verbal abuse (87%) (Shoghi et al. 2008). This is also congruent with another study, which was conducted to assess the violence in workplaces including health care setting, which stated that 80% of inpatients doctor and 91% of outpatient doctors underwent verbal abuse (Jankowiak et al. 2007) [20, 21]. Moreover, threat may precede the incidence of physical assault which put the victims in bad emotional status because of the terrifying episodes that they may experience during the period of threat [8].

An Australian study was concerned with the antecedents of violent actions toward nurses, which found that the majority of nurses (87% of 521) experienced violence in the past 6 months. Multiple factors were incorporated into occurrence of violent actions against nurses, which are presentation of the violent patients, and patient-specific behaviors. The former most common presentations were alcohol intoxication (84% of 491), mental health issues (77%), and substance abuse (76%), while the latter most common patient-specific factors were agitation (94% of 512), tone of voice (90%), attitude (88%), and pacing (87%) (Jacqueline V 2017) [23]. Constantly, the same results regarding the largest part of study’s sample being subjects to violence in the previous 6 months were found [24]. The effect of violence on nurses’ decision whether to resign or not was less in nurses with high burnout level. Moreover, higher rank nurses tend to respond violently and had a weaker intention to avoid violence. This attitude may be attributed to their longer experience and older age group that the nurses had (Yu-Fang 2018) [24].

### Limitations

This study encountered some limitations. First, involvement of only major hospitals in KSA and UAE, but this could be a result of absence of ED in rural areas and small cities in KSA or due to demographic reasons such as low population density areas in which they frequently seek health care services in major cities if critical injury occurred. In a survey of 700 respondents, 30% of our questionnaires was left blank. Another limitation is inability to collect data from the offenders regarding their mental health as a risk factor for committing violent action as was done by (Kowalenko T 2004) [25]. Also, trainees were not involved in this study to fill the questionnaires, which is an empty, ambiguous field for research needed to be conducted later. In order to obtain data from participants, our study depended on self-reporting which predispose our results to recall bias. English language is not considered a limitation in this study because health track education in both KSA and UAE is taught in English.

## Conclusion

Violent actions against ED staffs are common in gulf region wherein verbal abuses are committed as the most common form. These violent actions endanger HCW emotional stability rendering them in a state of burn-out. The exhibited failure to report such incidents should not be ignored by authorities and must be encouraged to deter further violations. Security measures should be escalated in hospitals where violent actions are frequently seen. Peace of mind and dodging involvement in social problems appeared to be the first priority for our health care providers.
